# Comparative performance of ISAGA IgM and ELISA assays for the diagnosis of maternal and congenital *Toxoplasma* infections: which technique could replace ISAGA IgM?[Fn FN1]

**DOI:** 10.1051/parasite/2024004

**Published:** 2024-02-08

**Authors:** Anne-Sophie Deleplancque, Hélène Fricker-Hidalgo, Christelle Pomares, Coralie L’Ollivier, Jean-Philippe Lemoine, Bernard Cimon, Luc Paris, Sandrine Houzé, Isabelle Villena, Hervé Pelloux, Odile Villard

**Affiliations:** 1 Centre National de Référence Toxoplasmose – Pôle Sérologie, Hôpitaux Universitaires de Strasbourg Strasbourg France; 2 CHU Lille, Parasitology Mycology Department, INSERM U1285, CNRS UMR 8576, Glycobiology in Fungal Pathogenesis and Clinical Applications, Université de Lille Lille France; 3 Laboratory of Parasitology and Mycology, Grenoble Alpes University Hospital and Institute for Advanced Biosciences, Grenoble Alpes University, INSERM U1209, CNRS UMR5309 Grenoble France; 4 Parasitology-Mycology laboratory, Côte d’Azur University, INSERM 1065, Nice University Hospital Nice France; 5 Centre Méditerranéen de Médecine Moléculaire (C3 M), U1065, Université Côte d’Azur, INSERM, Archimed Building 151 route Saint Antoine de Ginestière Nice France; 6 IHU-Méditerranée Infection, Assistance Publique Hôpitaux de Marseille (AP-HM) Marseille France; 7 Aix Marseille University, IRD, AP-HM, SSA, VITROME, IHU Méditerranée Marseille France; 8 Laboratoire de Parasitologie-Mycologie, CHU d’Angers Angers France; 9 Angers University, Brest University, IRF, SFR 4208 ICAT Angers France; 10 Parasitology laboratory, AP-HP Sorbonne Université, Hôpital Pitié-Salpêtrière Paris France; 11 Parasitology laboratory, AP-HP, Hôpital Bichat - Claude Bernard Paris France; 12 University of Paris Cité, IRD 261, MERIT Paris France; 13 Department of Parasitology and Medical Mycology, National Reference Centre on Toxoplasmosis, Reims Hospital Reims France; 14 Team EA 7510, SFR CAP-SANTE, Reims Champagne Ardenne University Reims France; 15 Institut de Parasitologie et de Pathologie Tropicale, UR7292 Dynamique des Interactions Hôte-Pathogène, Fédération de Médecine Transrationnelle, Université de Strasbourg Strasbourg France; 16 Laboratoire de Parasitologie et Mycologie Médicale, Hôpitaux Universitaires de Strasbourg Strasbourg France

**Keywords:** Toxoplasmosis, IgM detection, Sensitivity, Specificity, ISAGA IgM^®^

## Abstract

The ISAGA immunocapture test for the detection of anti-*Toxoplasma* immunoglobulin M is a manual technique known for its excellent sensitivity and specificity. The purpose of this retrospective, multicenter study was to compare the performances and agreement between ISAGA and other IgM detection techniques before cessation of ISAGA production. The analytic performance of the different tests was evaluated using 1,341 serum samples from adults with positive IgM and negative IgG to *Toxoplasma gondii*, and 1,206 sera from neonates born to mothers with seroconversion. The agreement between the tests was evaluated on 13,506 adult and 5,795 child serum samples. The sensitivity of Toxo-ISAGA IgM^®^ (adults 98.7%, neonates 63.1%) was similar to that of Platelia Toxo IgM^®^ (adults 94.4%, neonates 64.6%), and significantly higher than Liaison Toxo IgM^®^ (adults 90.6%), Architect/Alinity Toxo IgM^®^ (adults 95.7%, neonates 48.6%), and Vidas Toxo IgM^®^ (adults 81.8%, neonates 17.5%). However, the specificities varied between 24.4% (Platelia Toxo IgM^®^) and 95.2% (Liaison Toxo IgM^®^) in adults and were >95% for all tests in neonates. An analysis of the kappa coefficients showed better agreement between ISAGA IgM^®^ and the other tests in children (0.75–0.83%) than in adults (0.11–0.53%). We conclude that, in the absence of Toxo-ISAGA IgM^®^, the association of a very sensitive technique (Platelia Toxo IgM^®^ or Architect/Alinity Toxo IgM^®^) and a very specific technique (Vidas Toxo IgM^®^ or Liaison Toxo IgM^®^) is recommended for IgM detection in adult sera. For neonates, Platelia Toxo IgM^®^ appeared to be the best alternative to replace Toxo-ISAGA IgM^®^.

## Abstract (fr)

Le test d’immunocapture ISAGA pour la détection des immunoglobulines M anti-*Toxoplasma* est une technique manuelle connue pour son excellente sensibilité et spécificité. Le but de cette étude rétrospective et multicentrique était de comparer les performances et la concordance entre l’ISAGA et d’autres techniques de détection d’IgM avant l’arrêt de la commercialisation de l’ISAGA. Les performances analytiques des différents tests ont été évaluées à partir de 1 341 échantillons de sérum d’adultes présentant des IgM positives et des IgG négatives à *Toxoplasma gondii*, et de 1 206 sérums de nouveau-nés nés de mères présentant une séroconversion. La concordance entre les tests a été évaluée sur 13 506 échantillons de sérum d’adultes et 5 795 sérums d’enfants. La sensibilité de Toxo-ISAGA IgM^®^ (adultes 98,7 %, nouveau-nés 63,1 %) était similaire à celle de Platelia Toxo IgM^®^ (adultes 94,4 %, nouveau-nés 64,6 %) et significativement supérieure à celle de Liaison Toxo IgM^®^ (adultes 90,6 %), Architect/Alinity Toxo IgM^®^ (adultes 95,7 %, nouveau-nés 48,6 %) et Vidas Toxo IgM^®^ (adultes 81,8 %, nouveau-nés 17,5 %). Cependant, les spécificités variaient entre 24,4 % (Platelia Toxo IgM^®^) et 95,2 % (Liaison Toxo IgM^®^) chez les adultes et étaient >95 % pour tous les tests chez les nouveau-nés. L’analyse des coefficients kappa a montré une meilleure concordance entre ISAGA IgM^®^ et les autres tests chez les enfants (0,75–0,83%) que chez les adultes (0,11–0,53%). Nous concluons qu’en l’absence de Toxo-ISAGA IgM^®^, l’association d’une technique très sensible (Platelia Toxo IgM^®^ ou Architect/Alinity Toxo IgM^®^) et d’une technique très spécifique (Vidas Toxo IgM^®^ ou Liaison Toxo IgM^®^) est recommandée pour la détection des IgM dans les sérums adultes. Pour les nouveau-nés, Platelia Toxo IgM^®^ apparaît comme la meilleure alternative en remplacement de Toxo-ISAGA IgM^®^.

## Introduction

Primary infection by *Toxoplasma gondii* during pregnancy can result in congenital toxoplasmosis with possible severe fetal and neonatal complications [5, 7]. As the infection in pregnant women is asymptomatic in most cases, the diagnosis is based on serological results [11]. Specific immunoglobulin (Ig) M is produced during the first week after acute infection. Specific IgG is the last antibody to appear, a few weeks after IgM [4]. In a pregnant woman with negative IgG and positive IgM, the differential diagnosis between recent *T. gondii* infection and the presence of false IgM positivity is often difficult [6]. For *Toxoplasma* IgM detection, numerous automated immunoenzymatic or chemiluminescence tests [12] have been used, but a confirmatory test such as the IgM immunosorbent agglutination assay (ISAGA) is a help to determine whether the IgM are specific or not [2].

A positive diagnosis of primary *Toxoplasma* infection during pregnancy requires treatment of pregnant women until delivery, amniocentesis if possible, and serological and clinical follow-up of the neonate [8]. Serological follow-up of the neonate is based on the detection of IgM and/or IgA which do not cross the placenta, indicating congenital toxoplasmosis, as well as the detection of neosynthesized immunoglobulins by comparative mother–infant Western blot [3, 10]. The IgM ISAGA test is considered to be the method of choice for the detection of IgM in infants <6 months of age [9].

The ISAGA test is a manual technique with very good sensitivity and specificity [3]. Among the commercially available assays, ELISA and ELISA-like assays are commonly used as a first-line test. A second-line, more sensitive and specific assay, such as ISAGA, can be used if needed. Due to changes in European *in vitro* diagnostic regulations, the production and marketing of ISAGA will be stopped in 2024. The purpose of this study was to compare the performance of ISAGA with six automated IgM detection techniques to identify alternatives.

## Material and methods

### Ethics committee

This study was performed under the supervision of the French National Toxoplasmosis Reference Center, and benefited from agreements with the French Data Protection Agency (CNIL) and the ethics committee set up in order to implement and manage the National Reference Center on Toxoplasmosis database.

### Serum samples

This study involved a retrospective, multicenter analysis of results obtained from tests in 24 French referral centers for the diagnosis of toxoplasmosis. All referral centers were located in University Hospitals.

For the serum samples from adult patients, all centers searched their database and selected serological results with negative IgG and positive IgM by at least one technique. A total of 1,341 sera obtained from adult patients (one serum per patient) between 2018 and 2021 were classified into two groups: (i) the first group consisted of 156 serum samples with specific IgM obtained from patients with recent toxoplasmosis confirmed by the presence of IgG in further samples; (ii) the second group consisted of 1,185 sera from patients without toxoplasmosis but with non-specific IgM confirmed by the absence of IgG in further samples. In addition, the results of 13,506 adult serum samples tested by ISAGA IgM and other techniques were selected from the database of several centers and were used to calculate the agreement between ISAGA IgM and the other tests.

For the serum samples from neonates with suspected congenital toxoplasmosis, the centers searched their database between 2007 and 2017, and selected the results for 1,206 sera from neonates aged 0–10 days old. The sera from neonates were classified in two groups: (i) congenital toxoplasmosis; and (ii) no congenital toxoplasmosis, determined by molecular and serological assays. In addition, the results for 5,795 serum samples, collected from children <1 year of age between 2018 and 2021, were selected from the database of several centers and used to calculate the agreement between ISAGA IgM and the other tests.

### Serological techniques

All serum samples from adults with suspected seroconversion and from neonates with suspected congenital toxoplasmosis were analyzed using the immunosorbent agglutination assay, Toxo-ISAGA IgM^®^ (bioMérieux, Marcy-l’Etoile, France). Each sample was also analyzed using one or two immunoenzymatic or chemiluminescence tests to detect IgM. The techniques employed were the Liaison Toxo IgM^®^ assay (DiaSorin, Saluggia, Italy), Architect/Alinity Toxo IgM^®^ assay (Abbott Diagnostics, Wiesbaden, Germany), Vidas Toxo IgM^®^ assay (bioMérieux, Marcy l’Etoile, France), Platelia Toxo IgM^®^ assay (Bio-Rad, Marnes-la-Coquette, France), Cobas Toxo IgM^®^ assay (Roche Diagnostics, Basel, Switzerland or GmbH, Penzberg, Germany), and/or Advia Centaur/Atellica Toxo IgM^®^ assay (Siemens Healthcare Diagnostics, Deerfield, IL, USA). The results were interpreted using the cut-off for each test provided by the manufacturer.

### Data analysis

Confidence intervals (CIs) were calculated using the GraphPad QuickCalcs website: https://www.graphpad.com/quickcalcs/ConfInterval1.cfm (accessed November 2015). Multiple comparison tests were performed using GraphPad Prism version 8.0.0 for Windows (GraphPad Software, San Diego, CA, USA; https://www.graphpad.com). The agreement study was performed according to CLSI^®^ EP12-A2 recommendations. Estimates for sensitivity, specificity, and accuracy were computed according to the global interpretation of clinical and biological data (Wilson–Brown method) and a test of proportion (χ^2^ test: *p* < 0.05 was considered statistically significant). Actual agreement was analyzed by calculating Cohen’s kappa coefficient. Cohen’s kappa is widely used to show the overall observed agreement. Kappa = 1 if there is perfect agreement between the reagents and 0 if the observed agreement is equal to agreement expected by chance. It is considered to have perfect agreement between 0.81 and 1, good between 0.61 and 0.80, moderate between 0.41 and 0.60, fair between 0.21 and 0.40, and poor between 0.01 and 0.20 (interpretation of the strength of the agreement based on the Cohen’s kappa value [1]). The number of serum samples in the different groups was significantly different (*p* < 0.0001). This should be taken into consideration in the interpretation of the results.

## Results

### Suspected seroconversion in adult patients

Of the 156 sera from women with recent infection (IgM positive with one technique and IgG negative), 149 tested positive with Toxo-ISAGA IgM^®^, five were equivocal, and two were negative (Table 1). Of the 1,185 sera from women with non-specific IgM, 877 tested negative with Toxo-ISAGA IgM^®^, 154 were equivocal, and 154 were positive (Table 1). If borderline values were considered positive, the sensitivity, specificity, and accuracy of Toxo-ISAGA IgM^®^ were 98.7% (95.5–99.8), 74.0% (71.4–76.4), and 76.9%, respectively. If borderline values were considered negative, the sensitivity, specificity, and accuracy of Toxo-ISAGA IgM^®^ were 95.5% (91.0–97.8), 87.0% (85.0–88.8), and 88.0%, respectively. A comparison of the sensitivity, specificity, and accuracy was performed on sera analyzed by the same tests, because the performances varied depending on the serum groups assayed. Toxo-ISAGA IgM^®^ accuracy varied from 61.0% to 90.6% depending on the serum group (Table 2). The accuracy of the different techniques was better if borderline values were considered negative rather than positive. The sensitivity of Platelia Toxo IgM^®^ was similar to that of Toxo-ISAGA IgM^®^. However, the sensitivities of Architect/Alinity Toxo IgM^®^, Liaison Toxo IgM^®^, and Vidas Toxo IgM^®^ were significantly lower than that of Toxo-ISAGA IgM^®^. The specificities of Liaison Toxo IgM^®^ and Vidas Toxo IgM^®^ were significantly higher than that of Toxo-ISAGA IgM^®^. However, the specificity of Architect/Alinity Toxo IgM^®^ was significantly lower than that of Toxo-ISAGA IgM^®^. There was no significant difference between Platelia Toxo IgM^®^ and Toxo-ISAGA IgM^®^. The accuracy of Liaison Toxo IgM^®^ was higher than that of Toxo-ISAGA IgM^®^; in contrast, the accuracies of the other techniques were lower than that of Toxo-ISAGA IgM^®^.

**Table 1:** Results of IgM detection with Toxo-ISAGA IgM^®^, Liaison Toxo IgM^®^, Architect/Alinity Toxo IgM^®^, Vidas Toxo IgM^®^, and Platelia Toxo IgM^®^ assays on 1,341 adult sera with negative IgG and positive IgM by at least one test.

Clinical status	Assay	Number of samples with the following result		
		Negative	Equivocal	Positive
Recent *Toxoplasma* infection (seroconversion)	Toxo-ISAGA IgM^®^ (*n* = 156)	2 (1.3%)	5 (3.2%)	149 (95.5%)
	Liaison Toxo IgM^®^ (*n* = 96)	9 (9.4%)	6 (6.2%)	81 (84.4%)
	Architect/Alinity Toxo IgM^®^ (*n* = 47)	2 (4.2%)	2 (4.2%)	43 (91.5%)
	Vidas Toxo IgM^®^ (*n* = 11)	2 (18.2%)	2 (18.2%)	7 (63.6%)
	Platelia Toxo IgM^®^ (*n* = 18)	1 (5.5%)	1 (5.5%)	16 (88.9%)
Absence of *Toxoplasma* infection (nonspecific IgM)	Toxo-ISAGA IgM^®^ (*n* = 1,185)	877 (74.0%)	154 (13.0%)	154 (13.0%)
	Liaison Toxo IgM^®^ (*n* = 788)	719 (91.2%)	31 (3.9%)	38 (4.8%)
	Architect/Alinity Toxo IgM^®^ (*n* = 366)	221 (60.4%)	22 (6.0%)	123 (33.6%)
	Vidas Toxo IgM^®^ (*n* = 67)	48 (71.6%)	3 (4.5%)	16 (23.9%)
	Platelia Toxo IgM^®^ (*n* = 41)	10 (24.4%)	1 (2.4%)	30 (73.2%)

**Table 2:** Comparison of sensitivity, specificity, and accuracy of Toxo-ISAGA IgM^®^ and ELISA techniques (Liaison Toxo IgM^®^, Architect/Alinity^®^, Vidas Toxo IgM^®^, Platelia Toxo IgM^®^) on the same groups of adult sera.

Assay (number of sera)	Sensitivity % [95% CI]		Specificity % [95% CI]		Accuracy %		*P* value		
	Borderline values considered positive	Borderline values considered negative	Borderline values considered positive	Borderline values considered negative	Borderline values considered positive	Borderline values considered negative	Borderline values considered positive		Borderline values considered negative
Toxo-ISAGA IgM^®^ (*n* = 884)	97.9% [92.7–99.6]	94.8% [88.4–97.8]	90.1% [87.8–92.0]	78.7% [75.7–81.4]	80.8%	90.6%			
Liaison Toxo IgM^®^ (*n* = 884)	90.6% [83.1–95.0]	84.4% [75.8–90.3]	95.2% [93.5–96.5]	91.2% [89.0–93-0]	91.2%	94.0%	<0.0001	<0.0001	
Toxo-ISAGA IgM^®^ (*n* = 413)	100% [92.4–100]	93.6% [82.8–97.8]	70.2% [65.3–74.7]	85.8% [81.8–89.0]	73.6%	86.7%			
Architect/Alinity^®^ (*n* = 413)	95.7% [85.5–99.2]	91.5% [80.0–96.6]	60.4% [55.3–65.3]	66.4% [61.4–71.0]	64.4%	69.3%	<0.0001	<0.0001	
Toxo-ISAGA IgM^®^ (*n* = 78)	100% [74.1–100]	100% [74.1–100]	55.2% [43.4–66.5]	71.6% [59.9–81.0]	61.5%	75.6%			
Vidas Toxo IgM^®^ (*n* = 78)	81.8% [52.3–96.8]	63.6% [35.4–84.8]	71.6% [59.9–81.0]	76.1% [64.7–84.7]	73.1%	74.4%	0.0012	0.0126	
Toxo-ISAGA IgM^®^ (*n* = 59)	100% [82.4–100]	100% [82.4–100]	39.0% [25.7–54.3]	43.9% [29.9–59.0]	57.6%	61.0%			
Platelia Toxo IgM^®^ (*n* = 59)	94.4% [74.2–99.7]	88.9% [67.2–98.0]	24.4% [13.8–39.3]	26.8% [15.7–41.9]	45.8%	45.8%	0.1464	0.3068	

The results for sera analyzed by Cobas Toxo IgM^®^ and Advia Centaur^®^ or Atellica^®^ were studied separately because of bias in serum collection. The sera (*n* = 984) were sent by private medical laboratories to the referral laboratories to determine the presence of specific or nonspecific IgM. A total of 839 and 145 sera were positive or equivocal with Cobas Toxo IgM^®^ and Advia Centaur^®^/Atellica^®^, respectively (Table 3). Thirty sera were positive by Toxo-ISAGA IgM^®^ and follow-up confirmed toxoplasmosis. In the cases of nonspecific IgM, the Toxo-ISAGA IgM^®^ results (*n* = 812 sera positive with Cobas Toxo IgM^®^) were negative for 642 sera (79.1%), equivocal for 93 (11.5%), and positive for 77 sera (9.5%). In the cases of nonspecific IgM, the Toxo-ISAGA IgM^®^ results (142 sera positive with Advia Centaur^®^/Atellica^®^) were negative for 112 sera (78.9%), equivocal for 21 (14.8%), and positive for nine (6.3%).

**Table 3:** Results of IgM detection with Toxo-ISAGA IgM^®^ on 984 adult sera that were positive or equivocal with Cobas Toxo IgM^®^ (*n* = 839) or Advia Centaur/Atellica Toxo IgM^®^ (*n* = 145).

Clinical status	Assay (number of sera)	Number of samples with the following result by Toxo-ISAGA IgM^®^		
		Negative	Equivocal	Positive
Seroconversion	Cobas Toxo IgM^®^ (*n* = 27)	0	0	27
Seroconversion	Advia Centaur/Atellica Toxo IgM^®^ (*n* = 3)	0	0	3
Nonspecific IgM	Cobas Toxo IgM^®^ (*n* = 812)	642	93	77
Nonspecific IgM	Advia Centaur/Atellica Toxo IgM^®^ (*n* = 142)	112	21	9

This study of the agreement between Toxo-ISAGA IgM^®^ and Architect/Alinity Toxo IgM^®^, Vidas Toxo IgM^®^, Platelia Toxo IgM^®^, Liaison Toxo IgM^®^, and Atellica Toxo IgM^®^ was performed on 10,026, 2,123, 1,357, 6,683 and 142 sera, respectively (Table 4). Moderate agreement was observed between Toxo-ISAGA IgM^®^ and Vidas Toxo IgM^®^ (0.53%), Architect/Alinity Toxo IgM^®^ (0.52%), and Liaison Toxo IgM^®^ (0.41%), and poor agreement with Platelia Toxo IgM^®^ (0.12%) and Atellica Toxo IgM^®^ (0.11%).

**Table 4:** Cohen’s kappa coefficient and agreement in adults with positive Toxo-ISAGA IgM^®^ assay.

Assay	Number of sera	Agreement % ISAGA/other	Kappa coefficient [95% CI]
Liaison Toxo IgM^®^	6,683	73.3	0.41 [0.39–0.43]
Architect/Alinity Toxo IgM^®^	10,026	76.1	0.52 [0.50–0.53]
Vidas Toxo IgM^®^	2,123	76.7	0.53 [0.49–0.56]
Platelia Toxo IgM^®^	1,357	73.1	0.12 [0.08–0.16]
Atellica Toxo IgM^®^	142	41.6	0.11 [0.05–0.18]

### Suspected congenital toxoplasmosis in neonates

The 1,206 serum samples from neonates 0–10 days old included 715 sera (59.3%) from neonates with congenital toxoplasmosis and 491 (40.7%) from neonates without congenital toxoplasmosis. Out of the 715 sera from neonates with congenital toxoplasmosis, the Toxo-ISAGA IgM^®^ results were positive for 451 (63.1%) sera and negative for 264 (36.9%). Out of the 491 serum samples from neonates without congenital toxoplasmosis, the Toxo-ISAGA IgM^®^ results were negative for 483 (98.4%) sera and positive for 8 (1.6%), probably corresponding to maternal contamination. The sensitivity of Platelia Toxo IgM^®^ (64.6%) was similar to that of Toxo-ISAGA IgM^®^ (63.1%). In contrast, the sensitivities of Architect/Alinity Toxo IgM^®^ (48.6%) and Vidas Toxo IgM^®^ (17.5%) were significantly lower than that of Toxo-ISAGA IgM^®^. The specificities were >95% for the following four tests: Toxo-ISAGA IgM^®^ (98.4%), Platelia Toxo IgM^®^ (95.4%), Architect/Alinity Toxo IgM^®^ (100%), and Vidas Toxo IgM^®^ (100%) (Table 5).

**Table 5:** Comparison of sensitivity and specificity of Toxo-ISAGA IgM^®^ and ELISA techniques (Architect/Alinity^®^, Vidas Toxo IgM^®^, Platelia Toxo IgM^®^) on 1,206 neonate sera.

Assay (number of sera)	Sensitivity % [95% CI]	Specificity % [95% CI]	*p* value
Toxo-ISAGA IgM^®^ (*n* = 1206)	63.1% [59.5–66.5]	98.4% [96.8–99.2]	Reference
Architect or Alinity^®^ (*n* = 285)	48.6% [41.8–55.3]	100% [92.2–100]	<0.0001
Vidas Toxo IgM^®^ (*n* = 177)	17.5% [10.0–28.6]	100% [96.7–100]	<0.0001
Platelia Toxo IgM^®^ (*n* = 344)	64.6% [57.6–71.0]	95.4% [90.8–97.7]	0.2752

The agreement between Toxo-ISAGA IgM^®^ and Architect/Alinity Toxo IgM^®^, Vidas Toxo IgM^®^, Platelia Toxo IgM^®^, and Liaison Toxo IgM^®^ was investigated using 3,068, 750, 1,435, and 432 sera, respectively (Table 6). Perfect agreement was observed between Toxo-ISAGA IgM^®^ and Platelia Toxo IgM^®^ (0.83%) and Liaison Toxo IgM^®^ (0.83%), and good agreement with Vidas Toxo IgM^®^ (0.80%) and Architect/Alinity Toxo IgM^®^ (0.75%).

**Table 6:** Cohen’s kappa coefficient and agreement in children <1 year-old with the ISAGA assay.

Assay	Number of sera	Agreement % ISAGA/other	Kappa coefficient [95% CI]
Architect/Alinity Toxo IgM^®^	3,068	96.2%	0.75 [0.70–0.79]
Vidas Toxo IgM^®^	750	96.5%	0.80 [0.72–0.87]
Platelia Toxo IgM^®^	1435	96.7%	0.83 [0.78–0.88]
Liaison Toxo IgM^®^	432	96.8%	0.83 [0.74–0.92]

## Discussion

The detection of IgM is an important step in the diagnosis of toxoplasmosis in adults, especially pregnant women, and in neonates born to women infected during pregnancy. In pregnant women, the potential detection of nonspecific IgM makes it difficult to exclude a recent infection and may lead to serological misinterpretation [3]. The test used for confirmation of IgM positivity must have good specificity in order to differentiate the presence of specific or nonspecific IgM in adults. Furthermore, the confirmatory tests must have good sensitivity for the diagnosis of congenital toxoplasmosis in neonates [9]. In our study, the specificity observed on the sera of newborn less than 10 days old was excellent for all techniques (95.4–100%), and therefore the presence of IgM on the sera taken at 0–10 days of life due to maternal contamination by placental passage was exceptional.

In addition, the performance (sensitivity, specificity, and agreement) of the tests depends on the population studied and the chosen reference technique [12]. In our work, the presence or absence of toxoplasmosis in the two populations, adults and neonates, allowed us to calculate and compare the performance of the different tests. Our results revealed different performances in the two populations. Thus, replacement of Toxo-ISAGA IgM^®^ will be different depending on the population analyzed (adults or neonates). Moreover, toxoplasmosis serology is more than just IgM detection and a routine compromise between the levels of IgG and IgM detected should be found. Other techniques such as western blot are available to complete the diagnosis of toxoplasmosis in adults or neonates.

Among the automated tests, Cobas Toxo IgM^®^ and Advia Centaur^®^/Atellica Toxo IgM^®^ could not be studied under the same conditions as the other tests because of a bias in data selection. The evaluation concerned only the results for positive sera sent to the reference laboratories to confirm or rule out recent *Toxoplasma* infection. In cases with no toxoplasmosis, the results of Toxo-ISAGA IgM^®^ were negative for 79% of sera that were positive with Cobas Toxo IgM^®^ or Advia Centaur^®^/Atellica Toxo IgM^®^. These results demonstrate the importance of having a specific confirmatory test to avoid diagnostic errors.

Our study focused on automated immunoenzymatic or chemiluminescence tests to replace a manual technique, Toxo-ISAGA IgM^®^. However, Meroni *et al*. [6] demonstrated good performance of LDBIO-Toxo IgM Western blot to detect seroconversion (sensitivity 97.8%) and discriminate false-positive results (specificity 89.7%). No data are currently available concerning LDBIO-Toxo IgM Western blot for the diagnosis of congenital toxoplasmosis in neonates.

In the current study, an analysis of the kappa coefficients showed better agreement between ISAGA IgM^®^ and the other tests in neonates (0.75–0.83%) than in adults (0.11–0.53%). The sensitivity of Toxo-ISAGA IgM^®^ was identical to that of Platelia Toxo IgM^®^ and higher than that of the other tests in the two populations, in line with previous study data [12]. However, the specificities were >95% for all techniques in neonates and varied from 26% (Platelia Toxo IgM^®^) to 95% (Liaison Toxo IgM^®^) in adults. Platelia Toxo IgM^®^ seemed to have the performance that was closest to that of Toxo-ISAGA IgM^®^ and is the only test whose manufacturer recommends different dilutions depending on the age of the patient to be tested. Platelia Toxo IgM^®^ appears to be a good alternative to replace Toxo-ISAGA IgM^®^ in neonates because of its very good sensitivity. This finding confirms previous studies [2, 12]. Concerning adults, the accuracy of Liaison Toxo IgM^®^ was greater than that of Toxo-ISAGA IgM^®^, but another study [12] reported that Liaison Toxo IgM^®^ had lower sensitivity (61.7%) with a concordance of 51.7% with Toxo-ISAGA IgM^®^.

Specific IgM in adults could be detected using a combination of a very sensitive technique such as Platelia Toxo IgM^®^ or Architect/Alinity Toxo IgM^®^ and a very specific technique such as Vidas Toxo IgM^®^ or Liaison Toxo IgM^®^.
